# Research progress on exosomes in the otology field: a systematic review

**DOI:** 10.1186/s13287-025-04816-2

**Published:** 2025-12-01

**Authors:** Zhihong Hao, Chanyuan Zhang, Zewen Li, He Zhao, Yan Wang, Yifei Wang, Hanjing Wang, Dawei Liu, Limei Cui, Yan Sun

**Affiliations:** 1 School of Clinical Medicine, Shandong Second Medical University, Weifang, 261000 Shandong PR China; 2 Department of Otorhinolaryngology, Head and Neck Surgery, Yantai Yuhuangding Hospital, Qingdao University, Yantai, 264000 Shandong PR China; 3 Shandong Provincial Key Laboratory of Neuroimmune Interaction and Regulation, Yantai, 264000 Shandong PR China; 4 Shandong Provincial Clinical Research Center for Otorhinolaryngologic Diseases, Yantai, 264000 Shandong PR China; 5 Shandong Engineering Research Center for Precision Diagnosis and Treatment of Airway Diseases, Yantai, 264000 Shandong PR China; 6 Yantai Key Laboratory of Otorhinolaryngologic Diseases, Yantai, 264000 Shandong PR China; 7 Yantai Clinical Research Center for Otorhinolaryngologic Diseases, Yantai, 264000 Shandong PR China; 8 College of Life Sciences, Shandong Agricultural University, Taian, 271000 PR China; 9 School of Pharmacy, Shandong Technology Innovation Center of Molecular Targeting and Intelligent Diagnosis and Treatment, Binzhou Medical University, Yantai, 264003 PR China

**Keywords:** Exosomes, Biological foundations, Ear diseases

## Abstract

Exosomes, characterized by their distinctive structural and functional properties, are increasingly recognized as an important focus of research in otology, offering considerable promise for both diagnostic and therapeutic purposes. This review provides a systematic summary of the biological foundations of exosomes, with particular emphasis on their potential applications in the diagnosis and treatment of ear diseases. We begin by evaluating the translational utility of exosomes as biomarkers for the early detection of otologic diseases. We then explore the therapeutic mechanisms mediated by exosomes derived from mesenchymal stem cells (MSCs), various tissues, body fluids, and plant sources. Owing to their natural capacity to encapsulate biological cargo, exosomes represent highly promising delivery vehicles capable of efficiently transporting drugs, functional proteins, and nucleic acids to target sites within the ear, thereby offering innovative avenues for the treatment of otological conditions. Finally, this review outlines prospective research directions and critically examines the major scientific challenges that must be addressed to facilitate clinical translation.

**Graphical Abstract:**

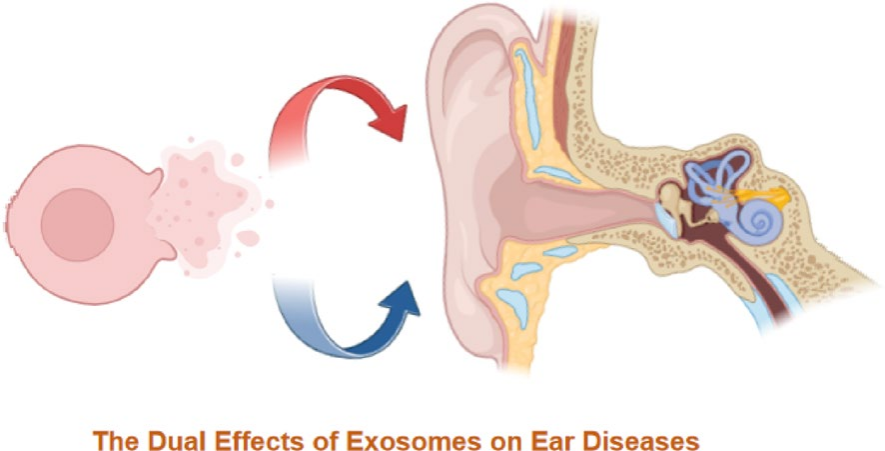

## Introduction

Ear diseases, encompassing a diverse array of conditions affecting the outer, middle, and inner ear, substantially impair patients’ quality of life and can lead to permanent hearing loss [1, 2]. Pathologies of the outer ear often involve structural abnormalities [3] or infections [4], middle ear disorders are commonly associated with infectious or functional issues, and inner ear diseases typically present as hearing loss or balance deficits [5, 6]. Conventional treatments largely depend on systemic or topical antibiotics [7] and invasive surgery [8, 9]. However, systemic drug delivery is frequently ineffective because of poor penetration through the blood–labyrinth barrier (BLB), resulting in subtherapeutic drug levels in the middle and inner ear and potential issue of antibiotics resistance [10, 11]. Invasive surgeries also carry risks such as inflammation or functional impairments. Moreover, curative therapies for diseases such as Meniere’s disease and sensorineural hearing loss (SNHL) are lacking [12, 13]. Current management practices often emphasize symptom control rather than remission and involve the use of pharmaceuticals or assistive devices to improve quality of life [14]. Clearly, effective treatments remain unmet for many otologic conditions, underscoring the urgent need to develop novel and efficacious therapeutic strategies.

Exosomes are small, membrane-bound extracellular vesicles (EVs) secreted by various cell types and serve as vital mediators of intercellular communication [15]. They carry and deliver bioactive molecules—including proteins, nucleic acids, and lipids—and possess a unique ability to cross biological barriers [15–17]. Recent years have witnessed an exponential increase in the number of research publications focused on exosomes, reflecting their value as an important modality for cell-to-cell signalling in diverse diseases, such as cancer [18, 19], and neurodegenerative [20, 21], cardiovascular [22, 23], hepatic [24], and pulmonary diseases [25]. In oncology, exosomes contribute to tumor progression by modulating cell proliferation, angiogenesis, and metastasis [26–28]. Specific molecules, such as exosomal miR-21, promote oncogenesis by binding to the 3’-UTR of target mRNAs and suppressing tumor suppressors such as programmed cell death 4 [29]. In neurodegenerative contexts, exosomes play dual roles [30, 31]. They can propagate inflammation and pathological proteins in Alzheimer’s disease (AD) and Parkinson’s disease (PD). For example, microglial activation plays regulatory roles in synaptic pruning [32], neuronal activity [33] and astrocyte transformation [34]. Preformed fibril-activated microglial exosomes can carry proinflammatory factors and toxic α-syn oligomers. These cargo components exacerbate neuroinflammation through the induction of a neurotoxic astrocyte phenotype and direct mediation of neuronal damage [35]. Conversely, exosomes can also confer protection by enhancing neuronal survival, clearing pathological proteins, and reducing neuroinflammation. For instance, in AD, neuronal exosomes enriched in glycosphingolipids can capture extracellular Aβ and facilitate its clearance by microglia [36].

Although the role of exosomes in disease mechanisms and their clinical potential have attracted broad attention in biomedical research, their implications in ear disorders have been less thoroughly explored. Existing evidence indicates that exosomes can deeply participate in the pathological process or therapeutic intervention of otological diseases through multiple mechanisms. The disease spectrum involved includes age-related hearing loss (ARHL) [37], ototoxic drug-induced hearing loss (OIHL) [38], SNHL [39], congenital malformations, and otitis media. The proposed mechanisms include regulating cell proliferation and apoptosis [40], modulating oxidative stress [41], and regulating inflammatory pathways [42]. In this review, we summarize the current biological understanding of exosomes and highlight their mechanistic and therapeutic potential across a spectrum of ear diseases. We first discuss the value of exosomes as diagnostic biomarkers in otology. We then focus on the therapeutic applications of exosomes derived from MSCs, tissues, body fluids, and plant sources. Additionally, we examine the promising role of exosomes as delivery vehicles for drugs, proteins, and nucleic acids in the treatment of ear diseases. Finally, we outline future research directions and ongoing challenges in the field. Although prior reviews have addressed the role of exosomes in targeted hearing loss treatment from the perspective of bioengineering technology [43], a comprehensive overview covering the full spectrum of ear disorders is lacking. This review aims to fill that gap by providing a detailed analysis of exosome biology and critically assessing their diagnostic, therapeutic, and clinical-translational value in otology.

## Search strategy and selection criteria

**Fig. 1:**
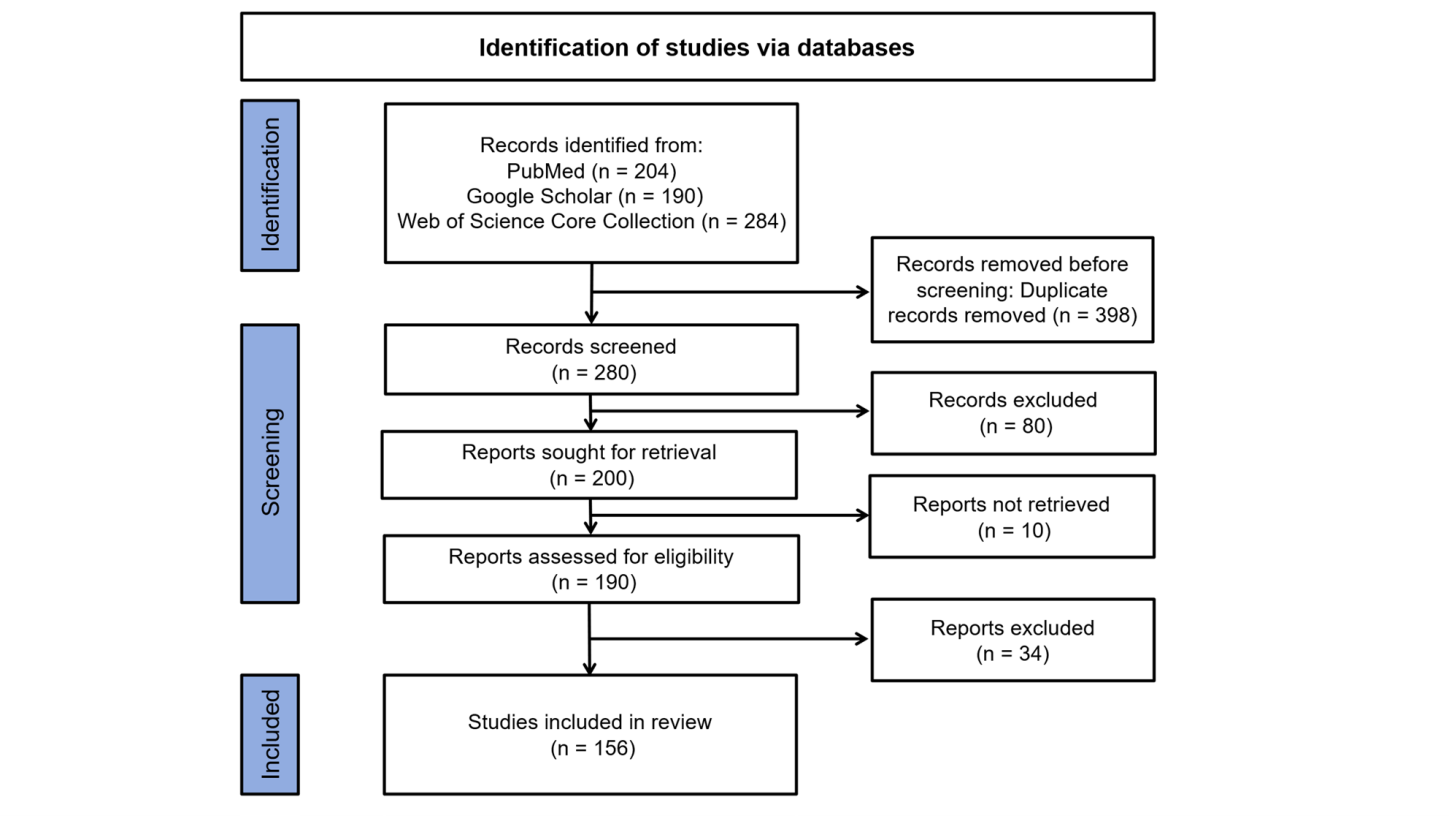
PRISMA flow chart

A comprehensive literature search was conducted across multiple electronic databases, including PubMed, Google Scholar and the Web of Science Core Collection. Search terms such as “otology”, “ear”, “auditory”, “hearing”, “hearing loss”, “deafness”, “exosomes”, “EVs”, and “extracellular vesicle” were used individually or in combination to query title, abstract, and keyword fields. No time restrictions were applied to ensure the inclusion of all relevant published studies. Retrieved records were screened on the basis of titles and abstracts to identify articles aligned with the objectives of this review. The final reference list was compiled according to the relevance of each study to the central topics discussed. Studies were included if they focused on the applications or mechanistic insights of exosomes in ear diseases. Studies were excluded if they did not involve exosomes or were not directly related to otological conditions. The study selection process is summarized in a Preferred Reporting Items for Systematic Reviews and Meta-Analyses (PRISMA) flow diagram (Fig. 1).

## Exosomes

**Fig. 2:**
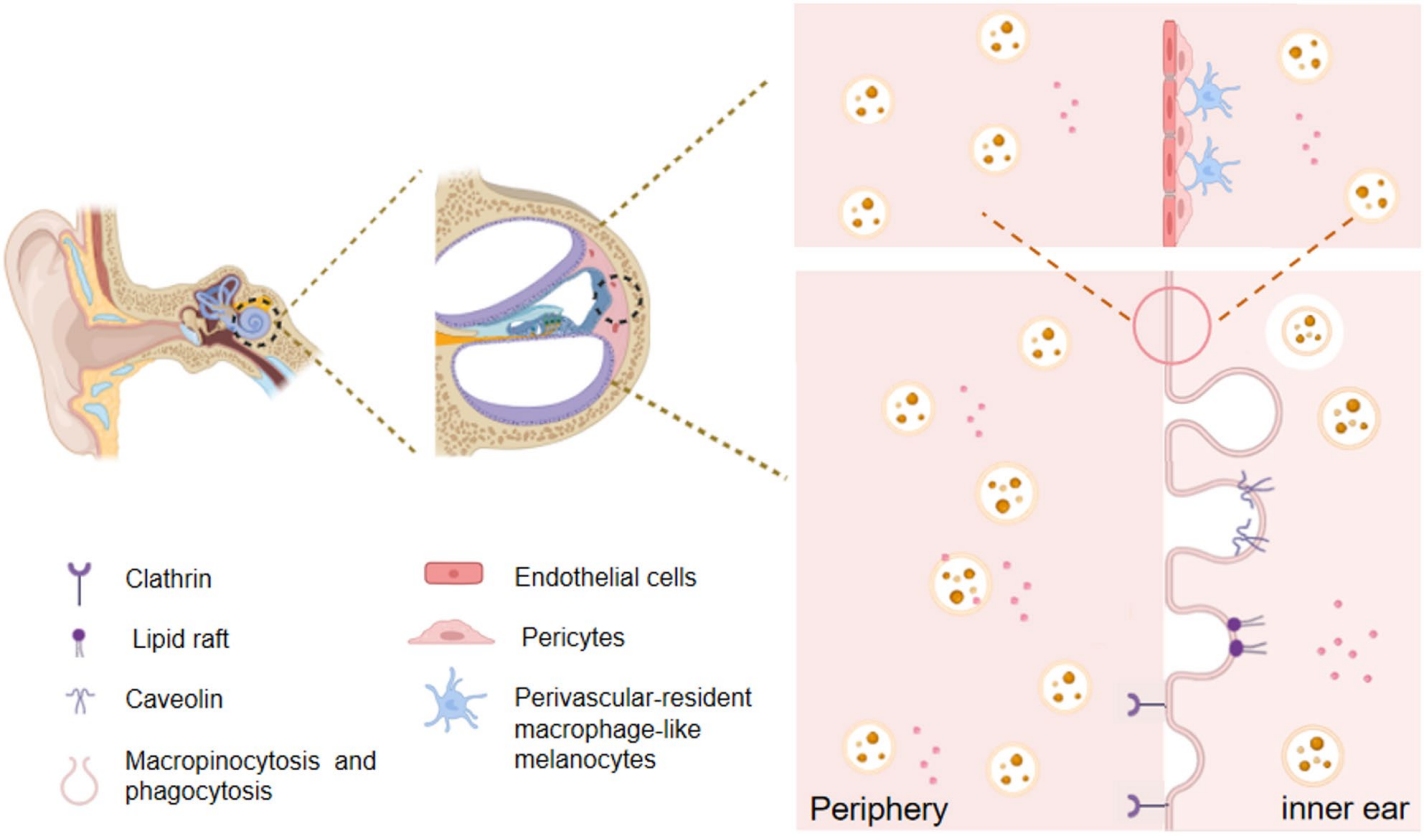
The anatomy of the ear and schematic diagram of exosomes crossing the BLB. The ear consists of the outer ear, the middle ear and the inner ear. The inner ear is mainly composed of the labyrinth system. The labyrinth includes the bony labyrinth and the membranous labyrinth. Stria vascularis is an important component of the inner ear, consisting of three layers, of which one layer is the BLB. The BLB is mainly composed of endothelial cells, pericytes, and perivascular-resident macrophage-like melanocytes surrounding the blood vessels, forming a selective permeability barrier. Exosomes enter the inner ear microvascular endothelial cells via different pathways, including clathrin-mediated, lipid raft mediated, caveolin- mediated, macropinocytosis and phagocytosis

Exosomes are membrane-bound vesicles released into the extracellular space through the fusion of multivesicular bodies (MVBs) with the plasma membrane [44]. They exhibit distinct structural and functional properties that render them highly promising for therapeutic applications. Structurally, exosomes are characterized by a lipid bilayer derived from the endocytic pathway [45], which encapsulates and stabilizes internal cargo, shielding it from enzymatic degradation and extracellular pH fluctuations [46]. With nanoscale diameters typically ranging from 30 to 150 nm, exosomes demonstrate enhanced tissue penetration and efficient diffusion, enabling effective delivery to target cells and tissues [47]. Their lumen contains a diverse array of biomolecules reflective of the parent cell, constituting a natural therapeutic payload. Importantly, the exosomal membrane can be engineered to improve targeting specificity and stability, further optimizing their function as delivery vehicles [48]. Functionally, exosomes exhibit low immunogenicity and high biocompatibility, reducing immune recognition and prolonging circulation time, thereby supporting safe and efficient cargo transport [49]. Furthermore, exosomes can traverse physiological barriers [50], such as the BLB (as depicted in Fig. 2). As pivotal mediators of intercellular communication, exosomes can effectively encapsulate and deliver a diverse spectrum of bioactive molecules, including drugs, proteins, and nucleic acids, while simultaneously providing protection against degradation in biological environments [51].

Exosome biogenesis is a highly regulated process involving endocytosis, the formation of early and late endosomes, the generation of intraluminal vesicles (ILVs) within MVBs, and the eventual release of exosomes upon MVB–plasma membrane fusion [52–55]. Early endosomes form at lipid raft regions of the plasma membrane and mature into late endosomes with the involvement of the Golgi apparatus. ILVs are generated through inward budding of the endosomal membrane. Although the mechanisms underlying cargo sorting into ILVs have not been fully elucidated, both the endosomal sorting complex required for the transport(ESCRT)–dependent and ESCRT–independent pathways are known to contribute to MVB formation [56, 57]. MVBs ultimately fuse with the plasma membrane, releasing their constituent ILVs into the extracellular environment as exosomes [58]. Tetraspanins such as CD9, CD63, and CD81, which are enriched on the exosomal membrane and are commonly used as exosomal markers, also play key roles in MVB biogenesis and exosomes release [59].

Exosomes can be derived from various sources, including tissues, bodily fluids, MSCs, and other cell types [60, 61]. However, their isolation remains methodologically challenging [62–64]. Differential ultracentrifugation (dUC), which separates particles on the basis of size and density using sequential centrifugation steps, is the most widely used approach and is often regarded as the gold standard [65–67]. Other commonly employed techniques include density gradient ultracentrifugation, ultrafiltration (UF), polyethylene glycol (PEG) precipitation, size-exclusion chromatography (SEC), immunoaffinity capture, nanoflow cytometry, microfluidic systems, and commercial kits [68, 69]. Each method presents distinct advantages and limitations: dUC is versatile and suitable for large sample volumes but is time-consuming and operator-sensitive and may yield relatively low purity [70]. PEG precipitation is simple and equipment-free but often coprecipitates impurities [71]. SEC preserves exosome integrity and offers high purity but has limited throughput, requires costly equipment, and may undersample smaller vesicles [72]. Immunoaffinity capture allows highly specific isolation of exosomes subpopulations but depends on antibody availability, is expensive, and is affected by marker heterogeneity [73]. Commercial kits facilitate straightforward processing without dUC, but the purity varies considerably across products [74]. It is also important to note that isolation techniques are continuously evolving, and current biomarkers may only detect specific exosomes subsets. Furthermore, storage conditions—particularly temperature and freeze‒thaw cycles—significantly influence exosomes stability and functionality, with − 80 °C generally recommended for long-term preservation [75].

## Exosomes as potential diagnostic markers

Exosomes carry a diverse cargo of biomolecules—including proteins, nucleic acids, lipids, and metabolites—and are present in various bodily fluids, such as blood and saliva. They facilitate intercellular communication and mirror the pathophysiological state of their parent cells, making them promising candidates for diagnosing ear diseases. Their inherent stability, biocompatibility, and ability to cross biological barriers such as the BLB further underscore their diagnostic potential in otology [76, 77].

Specific exosomal proteins have emerged as potential biomarkers for early detection in cancers [78] and other diseases [79, 80], including those of the ear. For instance, a 2018 study first identified exosomes in the inner ear, likely originating from the organ of Corti, suggesting that they could serve as indicators of inner ear physiology [80]. Ototoxic stress induced by cisplatin or gentamicin has been shown to alter both exosomal secretion and protein content. Among the most significantly upregulated proteins is Tmem33, whose expression is localized to the endoplasmic and nuclear membranes, although its functional role remains unclear [80]. Another multiomics study revealed that clathrin heavy chain (CLTC) in small extracellular vesicles (sEVs) derived from cisplatin-exposed cochlear explants may serve as a biomarker for drug-induced ototoxicity [38]. These findings imply that exosomal levels and contents are responsive to ototoxic damage and hold promise as novel biomarkers for inner ear pathology. Beyond inner ear diseases, exosomes also have diagnostic potential in middle ear diseases. For instance, patients with cholesteatoma exhibit higher concentrations of high-mobility group box 1 (HMGB1) carried by sEVs derived from plasma than are present in sEVs isolated from normal control plasma, suggesting their potential as a noninvasive biomarker for cholesteatoma [81].

Exosomal miRNAs also exhibit alterations in ear diseases. For example, compared with that in healthy controls, plasma-derived exosomal miR-93-3p is significantly elevated in SNHL patients, suggesting that it plays a role in pathogenesis and may be useful as a diagnostic biomarker [82]. In paediatric cases, middle ear effusions contain exosomes enriched in miRNAs, with miR-223-b being the most abundant—offering new avenues for early diagnosis and treatment [83].

**Fig. 3:**
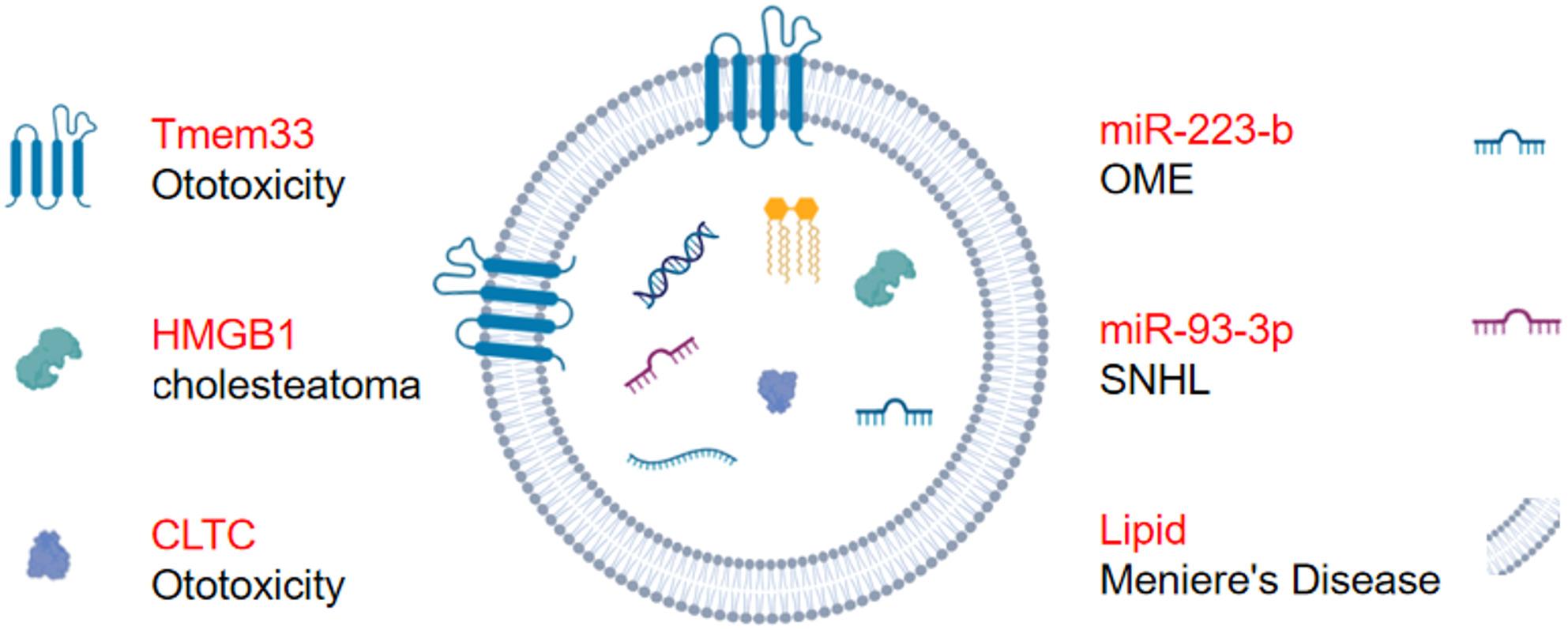
Exosomes as potential biomarkers in ear diseases. The Tmem33 carried by exosomes can serve as a sensitive indicator of ototoxic stress; HMGB1 can be a potential diagnostic marker for middle ear cholesteatoma; CLTC may be a potential biomarker for cisplatin-induced ototoxicity; miR-223-b can be a potential diagnostic marker for OME; miR-93-3p can be a potential diagnostic marker for SNHL; Changes in lipids composition can indicate compromised membrane integrity or cellular stress, and may be applied for the early detection of Meniere’s disease. Variations of cholesterol may indicate damage to the cochlea or vestibular structures

In addition to proteins and miRNAs, lipids present in exosomes may also serve as diagnostic biomarkers. Altered lipid profiles can indicate membrane instability or cellular stress [84], which may be relevant in conditions such as Meniere’s disease, which is often associated with endolymphatic hydrops [85]. Dysregulation of cholesterol metabolism, in particular, may reflect damage to cochlear or vestibular structures [86–88]. The distribution of these potential diagnostic biomarkers is summarized in Fig. 3.

## Exosomes as potential therapeutic molecules

Owing to their favourable biological properties, exosomes, which function as endogenous nanoscale messengers, have emerged as promising therapeutic agents for otological diseases. These advantageous properties include high biocompatibility [89], the ability to cross the BLB [90], and efficient delivery of bioactive molecules [79]. Notably, however, exosomes derived from different cellular sources display distinct biological features and mechanisms of action.

**Fig. 4:**
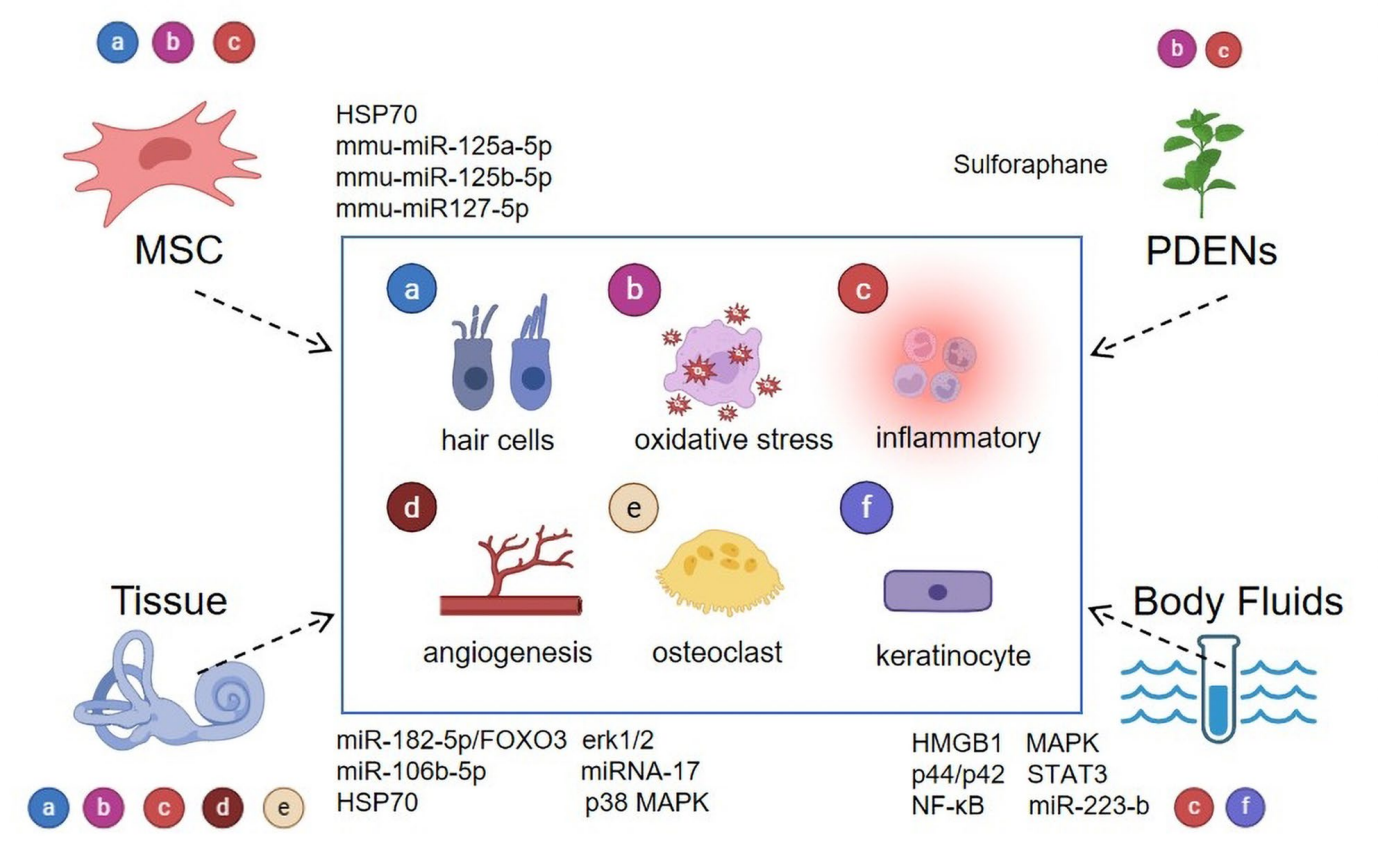
The specific mechanisms by which exosomes from different sources exert therapeutic or pathogenic effects in ear diseases. MSC-derived exosomes mainly target the damage and repair of hair cells, oxidative stress and inflammatory responses through key factors such as HSP70, mmu-miR-125a-5p, mmu-miR-125b-5p and mmu-miR127-5p. Tissue-derived exosomes mainly target the damage and repair of hair cells, oxidative stress, inflammatory responses, angiogenesis regulation and osteoclast through key factors such as miR-182-5p/FOXO3, miR-106b-5p, miRNA-17, HSP70, erk1/2 and p38 MAPK. Body Fluids-derived exosomes mainly target inflammatory responses and keratinocyte proliferation through key factors HMGB1, MAPK, p44/p42, STAT3, NF-κB and miR-223-b. PDENs mainly target oxidative stress and inflammatory responses through key factors such as Sulforaphane

For instance, exosomes from MSCs retain the multipotency and broad therapeutic potential of their parent cells. These MSC-derived exosomes markedly reduce the mRNA expression of proinflammatory cytokines—including IL-4, IL-23, IL-31, and TNF-α—thereby promoting an immunomodulatory microenvironment that supports inner ear repair [91]. Moreover, MSC-derived exosomes exhibit low immunogenicity, circumventing the risks associated with whole-cell transplantation, such as teratoma formation and vascular embolism [92, 93]. From a translational perspective, the scalable production of therapeutic exosomes can be achieved via three-dimensional culture systems combined with purification methods such as ultracentrifugation or SDS gel filtration chromatography [94, 95]. Tissue-derived exosomes carry molecular cargo reflective of their native microenvironment, offering insight into tissue-specific signalling and pathology [96, 97]. Exosomes derived from bodily fluids also hold clinical promise because of their minimally invasive isolation—which is compatible with routine clinical procedures [98, 99]—and advances in rapid microfluidic-based separation technologies [100, 101]. Finally, plant-derived exosome-like nanoparticles (PDENs) exhibit structural and compositional similarities to mammalian exosomes, and contain lipids, proteins, microRNAs, and metabolites that may facilitate cross-kingdom communication [102]. PDENs offer additional advantages such as cost-effective production, favourable safety profiles, and abundant sourcing, notably through organic farming [103], presenting a novel strategy for treating ear diseases. The diverse mechanisms through which exosomes originating from various sources can exert either therapeutic or pathogenic effects in the context of ear diseases are illustrated in Fig. 4. A summary of key studies on the role of exosomes from different sources in ear diseases is provided in Table 1.

### MSC-derived exosomes

MSC-derived exosomes, which can be isolated from multiple sources, such as adipose tissue and the umbilical cord, have been widely investigated owing to their lack of major ethical constraints, scalability [104, 105], and growing relevance in inner ear research.

These exosomes represent a promising therapeutic strategy for OIHL, with proposed mechanisms including suppression of inflammation, oxidative stress and immune modulation, etc. Preconditioning of MSCs—through heat shock or hypoxia—has been shown to increase the therapeutic potential of their exosomes. For instance, studies have indicated that MSC-derived exosomes mediated by heat shock protein 70 (HSP70) confer protection against cisplatin-induced ototoxicity in cochlear explants [106]. Yang et al. reported that exosomes from heat-shocked MSCs are enriched in HSP70 and attenuate cisplatin-induced ototoxicity by targeting the NOD-like receptor family pyrin domain containing 3(NLRP3) inflammasome, leading to preserved auditory function and reduced hair cell loss [107]. The authors highlighted HSP70-mediated pathways as a potential therapeutic strategy. Similarly, hypoxia-preconditioned MSC-derived exosomes upregulated hypoxia-inducible factor-1α, reducing oxidative stress and hair cell damage in a mouse model of cisplatin ototoxicity [41]. Another study combining RNA sequencing and proteomics demonstrated that MSC-derived exosomes mitigate hearing loss in mice by increasing the expression of glia-derived nexin (GDN) and specific miRNAs (e.g., mmu-miR-125a-5p, mmu-miR-125b-5p, and mmu-miR-127-5p), potentially inhibiting inflammation and protecting outer hair cells(OHCs) [108].

The therapeutic utility of MSC-derived exosomes extends beyond chemical ototoxicity to other auditory disorders. These vesicles exhibit strong neuroprotective properties, enhancing survival and neurite outgrowth in rat auditory neurons. Local application in the inner ear attenuated noise-induced hearing loss and hair cell damage, while in vitro assays confirmed the immunomodulatory effects on T cells and microglia [42]. Notably, in 2021, MSC-EVs were applied clinically as an adjunct to cochlear implantation in a patient with Meniere’s disease-related hearing loss. Speech intelligibility was maintained for 24 months without adverse effects, underscoring the translational potential of exosome-based therapies [109].

### Tissue-derived exosomes

Owing to their high tissue specificity, tissue-derived exosomes have demonstrated considerable potential for the treatment of both inner and middle ear diseases.

In inner ear disorders, exosome-based therapeutic strategies often focus on modulating inflammation, apoptosis, and oxidative stress. For instance, exosomes derived from cochlear spiral ganglion progenitor cells (CSGPC-exos), which are isolated from neonatal mouse cochlear explants, have been shown to alleviate cochlear ischaemia‒reperfusion (I/R) injury. These exosomes help preserve hearing thresholds in I/R-injured mice by downregulating proinflammatory cytokines (IL-6, IL-1β, TNF-α, and Cox-2) and upregulating anti-inflammatory miRNAs such as miR-21-5p, miR-26a-5p, and miR-181a-5p [110]. Lai et al. reported that inner ear stem cell-derived exosomes (IESCs-ex) reduce gentamicin-induced apoptosis and oxidative stress in House Ear Institute-Organ of Corti 1(HEI-OC1) cells through the miR-182-5p/FOXO3 axis [111]. Additionally, Breglio et al. identified a pathway in which cell-derived exosomes carrying HSP70 bind to Toll-like receptor 4 (TLR4) on hair cells, conferring protection against aminoglycoside-induced cell death [112]. Although the downstream survival signals activated by this interaction remain unclear, parallels can be drawn to cardiac protection mechanisms: plasma-derived exosomal HSP70 similarly binds TLR4 on cardiomyocytes, activating the ERK1/2 and p38 MAPK pathways and subsequently HSP27, thereby mitigating I/R injury [113, 114]. This suggests a conserved protective mechanism that may also operate in the inner ear. Notably, heat shock preconditioning enhances the release of HSP70-enriched exosomes from various cochlear cell types, including supporting cells and hair cells [115].

In middle ear pathologies, exosomes contribute to therapeutic outcomes through regulation of angiogenesis, bone remodelling, and inflammatory responses. For example, sEVs from human cholesteatoma perimatrix fibroblasts (hCPFs) exhibit reduced levels of miR-106b-5p. This downregulation increases angiopoietin 2 expression by alleviating miR-106b-5p-mediated suppression of its receptor, thereby promoting angiogenesis and potentially facilitating cholesteatoma progression [116]. Conversely, keratinocyte-derived exosomes (Ker-Exos) from cholesteatoma patients show decreased miRNA-17 expression, which may lead to increased receptor activator of nuclear factor-kB ligand(RANKL) levels in fibroblasts, stimulating osteoclast differentiation and bone resorption [117]. Furthermore, in an in vitro model of otitis media (OM), trophoblast-derived extracellular vesicles (TB-EVs) suppressed LPS-induced inflammation in human middle ear epithelial cells (HMEECs) in a concentration-dependent manner, reducing the levels of TNF-α, COX-2, and NF-κB [118], supporting the potential of EV-based therapies for mitigating middle ear inflammation.

### Body fluid-derived exosomes

Exosomes isolated from various bodily fluids, including lymph, plasma, and middle ear effusions (MEEs), display diverse therapeutic potential for middle and inner ear disorders. Their mechanisms of action and clinical applicability depend on both their cellular origin and the specific pathological context.

Using immunomagnetic nanoparticle technology, Zhuang et al. were the first to isolate exosomes from human perilymph (2–5 µL) collected during surgery in patients with SNHL [119]. This study provided the first conclusive evidence of exosomes in human perilymph and revealed the presence of hair cell markers such as myosin VIIa, suggesting that these vesicles originate at least partially from auditory or vestibular hair cells. These findings imply that hair cell-derived exosomes may modulate the inner ear microenvironment, with dysregulated release or modified cargo composition potentially contributing to SNHL pathogenesis.

In studies on cholesteatoma, compared with controls, sEVs isolated from patient plasma presented elevated levels of HMGB1. These HMGB1-enriched sEVs promoted keratinocyte proliferation and the secretion of proinflammatory cytokines such as IL-6, likely facilitating cholesteatoma progression through the activation of the MAPK p44/p42, STAT3, and NF-κB signalling pathways [81].

Furthermore, exosomes enriched with miRNAs have been identified in the MEE of paediatric patients. One study detected 29 MEE-specific miRNAs, among which miR-223-b—a neutrophil-associated miRNA—was the most abundant [83]. Proteomic characterization further revealed that these vesicles carry substantial amounts of both miRNAs and proteins, indicating that they are important mediators of innate immune responses and neutrophil functions. These findings provide new insights into exosome-mediated intercellular communication and suggest novel mechanisms underlying the pathophysiology of otitis media.

### PDENs

In recent years, PDENs have gained increasing attention as promising alternatives to mammalian cell-derived exosomes [120]. PDENs are rich in diverse bioactive compounds and exhibit considerable potential to modulate inflammatory responses, oxidative stress, and immune function [121, 122]. For instance, exosomes isolated from *Citrus limon* have been shown to regulate macrophage polarization and promote tissue regeneration [123], suggesting their therapeutic applicability in inflammatory ear conditions such as otitis externa and media. Similarly, PDENs derived from eggplant berries demonstrate notable anti-inflammatory effects, including the suppression of IL-6 production in LPS-stimulated RAW264.7 macrophages [124].

In addition to their role in inflammatory disorders, PDENs also show promise in the treatment of hearing loss, which often involves oxidative stress and inflammation leading to sensory cell damage. Various insults—such as radiation, ageing, cochlear implantation trauma, noise exposure, and ototoxic drugs—can induce reactive oxygen species production in the inner ear, ultimately resulting in hearing impairment [125–127]. Targeting immune and oxidative pathways using exosome-based approaches represents an emerging strategy for hearing protection. Sulforaphane, a well-characterized antioxidant abundant in cruciferous vegetables, has been shown to attenuate cisplatin-induced hearing loss [128] and suppress schwannoma growth in vivo [129]. Notably, exosomes derived from broccoli may contain sulforaphane and serve as a natural, antioxidant-rich therapeutic vehicles for hearing preservation. Further mechanistic studies elucidating the anti-inflammatory, antioxidant, and immunomodulatory properties of PDENs are essential to fully exploit their biomedical potential in otology.

**Table 1:** Summary table of key studies on exosomes from different sources in otologic diseases

Source of exosomes	Exosome isolation methods	Experimental model	Handling method	Key factor	Disease	References
MSC-EVs	-	C57BL/6 mice	Intratympanic administration	-	NIHL	[42]
MSC-EVs	Isolation reagent	The cochlear explants	Co-cultures	HSP70	OIHL	[106]
MSC-Exo	Ultracentrifugation	C57BL/6 mice	Trans-tympanical injection	HSP70	OIHL	[107]
MSC-Exo	Ultracentrifugation	C57BL/6 mice	Trans-tympanically administered	-	OIHL	[41]
Supporting cells	Ultracentrifugation	Utricle culture	-	HSP70	OIHL	[112]
MSC-Exo	Filtering	C57BL/6 mice	The round window niche injection	mmu-miR-125a-5p, mmu-miR-125b-5p and mmu-miR127-5p	OIHL	[108]
IESCs-ex	Differential ultracentrifugation	The HEI-OC1 cell line	-	miR-182-5p/FOXO3	OIHL	[111]
CSGPC-exos	Density gradient centrifugation	C57BL/6 mice	-	-	Idiopathic sudden sensorineural hearing loss	[110]
hCPFs-Exo	Differential ultracentrifugation	CPFs and SFs	Co-cultures	miR-106b-5p	Cholesteatoma	[116]
Ker-Exo	Differential ultracentrifugation	Cholesteatoma keratinocytes and fibroblasts	Co-cultures	miRNA-17	Middle ear cholesteatoma	[117]
plasma-derived sEVs from cholesteatoma patients	SEC	human keratinocytes	Treatment of cell lines with sEVs	HMGB1	Middle ear cholesteatoma	[81]
TB-EVs	Ultracentrifugation	In vitro inflammation model in HMEECs by LPS	Co-cultures	-	OM	[118]
MEE	The Exoquick-TC kit	-	-	miR-223-b	OM	[83]

MSC-EVs: Mesenchymal stem cell-derived extracellular vesicles; MSC-Exo: Mesenchymal stem cell-derived exosomes; NIHL: Noise-induced hearing loss; OIHL: Ototoxic drug-induced hearing loss; HEI-OC1: House Ear Institute-Organ of Corti 1; IESCs-ex: exosomes derived from Inner ear stem cells; CSGPC-exos: Cochlear spiral ganglion progenitor cell-derived exosomes; hCPFs-Exo: Human cholesteatoma perimatrix fibroblast-derived exosomes; CPFs: Cholesteatoma perimatrix fibroblasts; SFs: Stromal fibroblasts; Ker-Exo: Keratinocyte-derived exosomes; SEC: Size-exclusion chromatography; sEVs: Small extracellular vesicles; TB-EVs: Trophoblast-derived extracellular vesicles; HMEECs: Human middle ear epithelial cells; OM: Otitis media; LPS: Lipopolysaccharide; MEE: Middle Ear Effusion; TC: Tissue Culture

## Exosomes as delivery vehicles

The complex anatomy of the inner ear, particularly the specialized BLB, substantially impedes systemic drug delivery [130, 131], presenting a major obstacle for treating inner ear disorders such as SNHL, Meniere’s disease, and noise-induced hearing loss (NIHL). Although local delivery approaches, including intratympanic injection [132] and drug-eluting cochlear implants [133], offer alternative routes, they carry risks of iatrogenic injury and subsequent inflammatory responses [134]. Therefore, targeted delivery of otoprotective agents directly to the inner ear has long been regarded as an ideal strategy for hearing preservation and restoration.

**Fig. 5:**
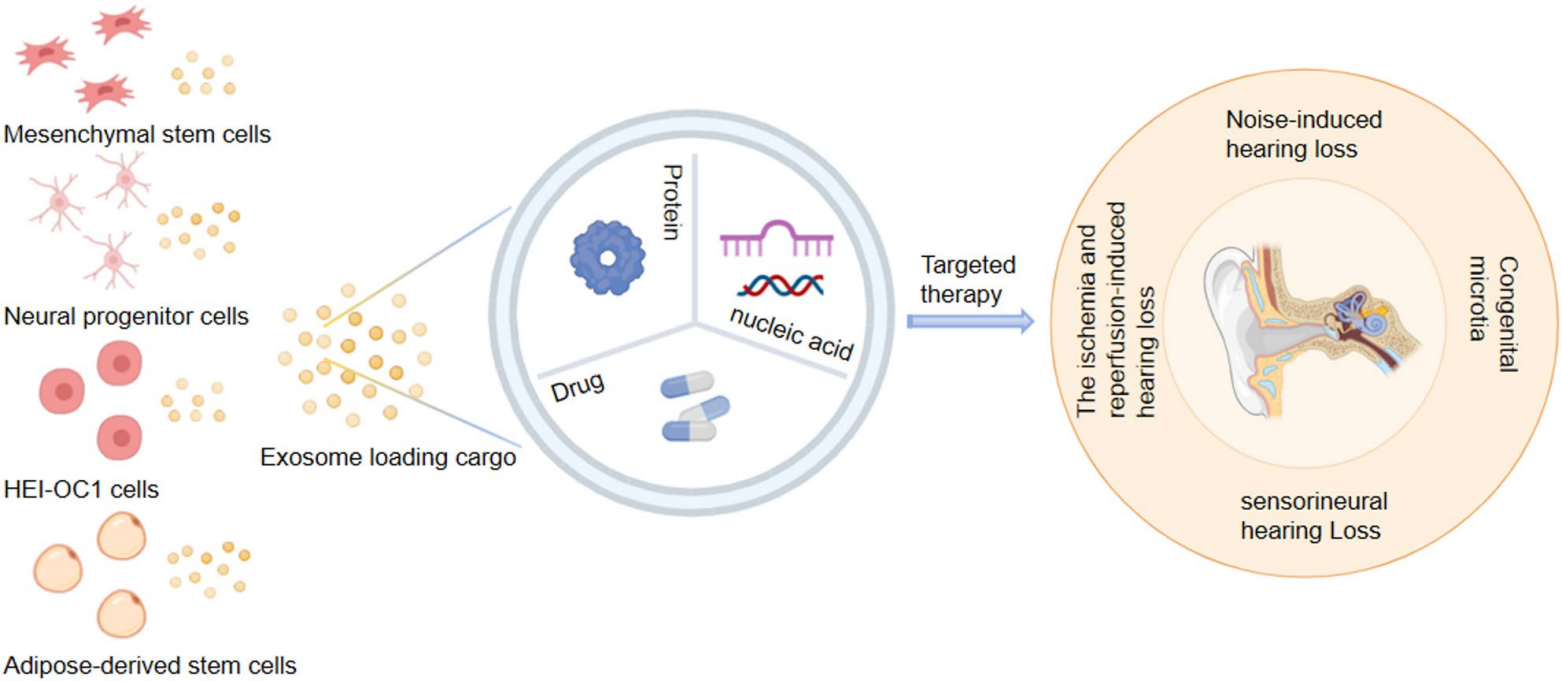
Exosomes exert potential therapeutic effects in ear diseases by delivering different therapeutic molecules

As natural nanocarriers, exosomes have emerged as promising vehicles for this purpose. They demonstrate intrinsic protective properties and a native ability to target cochlear and vestibular hair cells [90]. Key advantages include high biocompatibility, low immunogenicity, substantial drug-loading capacity, the ability to cross biological barriers such as the BLB, and functional programmability [135–137]. These features have spurred growing interest in their application as delivery platforms in otological therapeutics. Exosomes can be loaded with diverse therapeutic agents, including small-molecule drugs, proteins, and nucleic acids. The potential therapeutic mechanisms of exosomes from various sources in treating ear diseases through targeted molecular delivery are shown in Fig. 5, and the key parameters of exosome-based delivery systems for otological applications are summarized in Table 2.

### Delivery of medications

Current clinical management of ear diseases primarily involves the use of antibiotics (e.g., levofloxacin ear drops, cefdinir, amoxicillin) and glucocorticoids administered either locally or systemically [138, 139]. Although topical ear drops are noninvasive, their ability to cross the tympanic membrane and reach middle and inner ear structures is often limited [140]. Intratympanic injections allow direct drug delivery but are invasive, carry risks of infection and tympanic membrane injury, and are not well suited for repeated treatments [141, 142]. Systemic administration, on the other hand, is hindered by the BLB, which restricts drug penetration and results in low inner ear bioavailability [143, 144].

Exosomes, which are natural nanoscale vesicles, represent a promising alternative delivery system because of their ability to cross the BLB, inherent target specificity, high biocompatibility, and protective capacity for encapsulated therapeutics [49, 145]. The HEI-OC1 cell line—an immortalized human inner ear-supporting cell line—exhibits intrinsic homologous targeting properties [146]. Recent studies have demonstrated that both sEVs, including exosomes, and large EVs derived from HEI-OC1 cells can serve as nanocarriers for anti-inflammatory drugs and mediators such as Annexin A1 and galectins-1 [147]. Moreover, Kalinec et al. demonstrated that HEI-OC1-derived EVs can be loaded with pharmacological agents such as dexamethasone via simple incubation, supporting their utility as versatile nanocarriers for targeted molecule and drug delivery to the inner ear [148].

### Delivery of proteins

Proteins, as central executors of cellular functions, represent promising therapeutic agents capable of rapidly compensating for cellular deficits or enhancing functional pathways through direct delivery [149, 150]. Exosomes—nanoscale vesicles naturally released by cells—serve as effective vehicles for protein transport. They can encapsulate a wide variety of proteins, and techniques such as ultrasound microfluidics further improve their loading efficiency [151, 152]. The exosomal membrane also provides protection against enzymatic degradation, enhancing the stability and functional duration of encapsulated proteins in biological environments [153].

Brain-derived neurotrophic factor (BDNF), a key member of the neurotrophin family, plays essential roles in synaptic plasticity and neuronal survival in both the peripheral nervous system and central nervous system [154, 155]. BDNF has been shown to have protective effects on ribbon synapses and cochlear nerve fibres when it is administered locally via the round window membrane [156]. However, its large size prevents efficient transit across the BLB, limiting its systemic use for inner ear therapy [157]. Recent studies have turned to MSC-sEVs as nanocarriers to overcome this constraint. For example, systemically administered BDNF-loaded sEVs (BDNF-sEVs), delivered via tail vein injection, conferred significant protection in an NIHL mouse model [158]. These BDNF-sEVs reduced hair cell loss, preserved auditory function, and attenuated damage to inner hair cell ribbon synapses and cochlear nerve endings—effects further validated in cochlear explants [158]. In a separate study utilizing a Shaker-1 mouse model of dominant progressive hearing loss, a novel high-throughput microfluidic droplet electroporation system (µDES) facilitated the effective delivery of gRNA: Cas9 ribonucleoprotein (RNP) complexes to inner ear hair cells to engineer EVs [159]. This strategy led to a significant reduction in Myosin 7a^sh1^ mRNA expression and substantial hearing recovery, as measured by the auditory brainstem response (ABR).

### Delivery of nucleic acids

Nucleic acid therapeutics represent a promising strategy for treating genetic and acquired hearing disorders by directly targeting pathogenic genes or modulating relevant molecular pathways. Although adeno-associated virus (AAV) is regarded as the most promising carrier for cochlear gene delivery at present, its clinical application still faces multiple challenges. These include the low transduction rates of OHCs in the organ of Corti and vestibular hair cells in adult mice [160], as well as the need for further assessment of its biological safety [161]. To overcome these limitations, researchers have focused on exosomes and isolated exosome-associated AAV (Exo-AAV) from the culture medium of vector-producing 293T cells. Studies have shown that compared with traditional AAV, which can specifically target only inner hair cells (IHCs), Exo-AAV vectors exhibit outstanding transduction capabilities and can efficiently mediate the precise delivery of the Lhfpl5 gene to IHCs, OHCs, and vestibular hair cells in the cochlea both in vitro and in vivo [162]. Notably, this composite vector not only significantly improves gene transduction efficiency but also shows good tolerance.

Furthermore, exosomes can function independently as nucleic acid delivery vectors and play a positive role in ear diseases. For example, intracochlear administration of miR-21-enriched exosomes derived from neural progenitor cells (miR-21-Exo) attenuated hearing threshold shifts at 8, 16, and 32 kHz in I/R injury models. Mechanistically, miR-21-Exo upregulated IL-10 while suppressing TNF-α and IL-1β expression at both the transcriptional and protein levels, thereby ameliorating I/R-induced hearing loss [163].

Microtia, a congenital malformation of the auricle, can profoundly affect psychosocial development in children. Tissue-engineered cartilage constructs represent a promising alternative for ear reconstruction [164, 165]. In this context, Chen et al. developed engineered exosomes enriched with hsa-mir23a-3p using passage 4 adipose-derived stem cells (ADSCs) as parent cells. These exosomes promoted cell proliferation, reduced apoptosis, and enhanced cartilage regeneration, indicating their utility in engineered ear cartilage [40]. The same team also established an efficient method for isolating chondrocyte-homing peptide-modified extracellular vesicles (CHP-EVs) from 3D dynamic cultures of hADSC spheroids. These CHP-EVs penetrated biomaterial scaffolds and delivered therapeutic miRNAs specifically to residual chondrocytes, modulating local M1 macrophage infiltration and enhancing chondrocyte proliferation while reducing apoptosis—ultimately improving cartilage formation [166].

**Table 2:** A summary table of parameters of exosomes delivery systems in the treatment of ear diseases

Vehicle	Load mode	Deliver goods	Mechanism	Result	Sample size	Limitation	Reference
MSC-sEVs	Lentiviral transfection	BDNF	By attenuating oxidative stress and apoptosis.	BDNF-enriched MSC-sEVs afford substantial protection of hair cells, inner hair cell ribbon synapses, and cochlear nerve terminals against noise exposure	*n* = 5 animals/group.	The pharmacoki netics and in vivo distribution of BDNF-sEVs need further investigation	[158]
NPCs-Exo	Lentiviral transfection	miR − 21	Regulates the transcription of inflammatory cytokines.miR-21-Exo administration could diminish the upregulated expression of P53.	miR-21-Exo alleviates hearing loss.miR-21-Exo reduces hair cell apoptosis Induced by I/R.	*n* = 7	-	[163]
HEI-OC1-derived EVs	Loading was performed by co-incubation for 1 h at 25℃ with sonication for 5 min	Anti-inflammatory drugs and pro-resolution mediators	-	Auditory HEI-OC1 cells generate abundant EVs, both small (< 150 nm diameter) and larger (>150 nm diameter), which can be loaded with anti-inflammatory drugs, polyunsaturated fatty acid, and pro-resolving mediators, either alone or combined, in amounts significantly higher than those normally found in human serum.	-	Nevertheless, experiments aimed specifically at comparing Small-EVs and Large-EVs drug delivery to particular organ or cell targets must be performed before arriving at a definitive conclusion.	[147]
HEI-OC1-derived EVs	Co-incubation	Dexamethasone	-	HEI-OC1 cells are able to easily incorporate dexamethasone	-	Nanosight measurements are strongly dependent on the optical properties of the particles, which vary with their size. This problem is particularly evident in experiments such as those reported here, where the samples include particles in a very wide size range (50–800 nm).	[148]
HEI-OC1-derived EVs	By leveraging a novel high-throughput µDES	gRNA: Cas9 RNP complex	RNP EVs can disrupt the expression of Myo7ash1 allele at the mRNA level, thereby attenuating the oxidative stress that is associated with progressive hearing impairment.	The effective delivery of RNP-EVs into inner ear hair cells, with a clear reduction of Myo7ash1 mRNA expression compared to RNP-loaded lipid-like nanoparticles, leading to significant hearing recovery measured by ABR.	(*n* = 6)	It is still challenging to predict the gradual or drastic shifts on the function of final in-frame mutants based on the computational toolkits	[159]
Exo - AAV	-	Lhfpl5	Exo-AAV outperforms conventional AAV in transgene delivery to cochlear hair cells	Partially rescues hearing in a mouse model of hereditary deafness	(*n* = 38 for cochleostomy; *n* = 23 for round window membrane)	The window of therapeutic intervention is limited for Lhfpl5. not test distortion product otoacoustic emissions	[162]
ADSCs-3D-Exo	Lipofectamine 3000	hsa-miR-23 a-3 p	Enhancing cell proliferation and attenuating apoptosis. 3D-Exo activated the PTEN/PI3K/AKT/mTOR axis of microtia chondrocytes through delivering hsa-miR-23a-3p	Enhance the proliferation, survival and mature cartilage formation of microtia chondrocytes	-	Need to further evaluate the effects of engineered-exosome on the elastin composition and elasticity characteristics of the tissue-engineered cartilage. the release ratio of exosomes should be further enhanced.	[40]

EVs: extracellular vesicles; MSC-sEVs: Mesenchymal stem cell-derived small extracellular vesicles; BDNF: Brain-Derived Neurotrophic Factor; NPCs-Exo: neuroprogenitor Cell-derived Exosomes; I/R: Ischemia-reperfusion; HEI-OC1: House Ear Institute-Organ of Corti 1; µDES: Microfluidic droplet electroporation system; RNP: Ribonucleoprotein; gRNA: Guide RNA; Cas9: CRISPR-associated protein 9; ABR: auditory brainstem response; Myo7a: Myosin 7a; Exo - AAV: Exosome-associated Adeno-associated Virus; AAV: Adeno-associated virus; ADSCs: adipose-derived stem cells; ADSCs-3D-Exo: Adipose-derived stem cell 3D culture-derived exosomes

## Prospects and challenges

Although exosomes were first described in the 1980s [167], their identification and characterization in the inner ear were not reported until 2018 [80]. Since then, research on exosomes in the context of otological diseases has expanded rapidly, reflecting the field’s novelty and dynamic growth while revealing numerous promising avenues for future investigation.

Owing to their nanoscale size, exosomes can traverse intricate intercellular spaces within tissues, facilitating intercellular communication and biomolecule transfer that modulate complex signalling pathways. Their ability to function through autocrine and paracrine mechanisms makes them suitable candidates for engineered targeted delivery of therapeutic cargo. For example, lipidomic profiling of EVs derived from HEI-OC1 cochlear cells revealed elevated levels of anti-inflammatory and proresolving ceramide species compared with those in human serum, suggesting a role in immunomodulation within the auditory microenvironment [147]. Building on in vitro evidence that recombinant hydrogen peroxidase derivatives protect against cisplatin-induced ototoxicity, a macrophage-derived exosome system has been proposed for peroxisomal delivery, potentially enhancing otoprotective efficacy in vivo [168]. These findings underscore the multifunctional nature of exosomes and provide a foundation for innovative treatments for hearing disorders.

When delivered via exosomes, HSP70 has been demonstrated to have protective effects against ototoxic drug-induced hearing loss. The mechanism involves the binding of HSP70 to TLR4 on hair cells [112], leading to the activation of the ERK1/2 and p38 MAPK pathways and the subsequent upregulation of HSP27, which inhibits the apoptotic signalling induced by aminoglycosides. HSP70 may also suppress the NLRP3 inflammasome, attenuating inflammatory responses following cisplatin exposure [107]. This targeted exosome-mediated approach does not enable precise drug delivery across the blood–labyrinth barrier but also represents a promising strategy against aminoglycoside- and cisplatin-induced ototoxicity.

Despite the considerable progress in exosomes research, substantial challenges remain. The delicate and microscopic nature of the inner ear necessitates highly purified exosomes that must be delivered with precision and at effective doses. The purification of exosomes, a critical step for clinical translation, is hampered by technical limitations. Current mainstream separation techniques, such as ultracentrifugation and immunoaffinity chromatography, suffer from drawbacks, including operational complexity, low yield, and difficulty in scaling up for large-scale production [169, 170]. The absence of standardized protocols, coupled with the reliance on specialized equipment and expertise, further increases costs [171, 172]. Moreover, the inherent size heterogeneity within the exosomes population impedes the isolation of homogenous subgroups, potentially impacting subsequent applications [173, 174]. Targeted delivery strategies, crucial for precise drug delivery to lesion sites, also face considerable obstacles. Native exosomes exhibit limited tissue-specific targeting [175], leading to potential off-target accumulation in nontargeted tissues within the ear microenvironment, such as adjacent nerves or vascular systems. This condition can compromise treatment accuracy and induce adverse effects.

Consequently, the development of simplified, efficient, safe, and high-yield methods for producing engineered exosomes is paramount for improved clinical outcomes. Engineered modifications, such as surface ligand conjugation or therapeutic cargo loading (e.g., anti-inflammatory factors, neurotrophic factors, or miRNAs), can enhance the targeting capabilities or therapeutic potential of exosomes [176]. However, new challenges, including drug-loading strategies such as electroporation or ultrasound-assisted methods, can compromise exosomes integrity and activity, leading to reduced overall efficiency. Furthermore, modified exosomes may elicit immune responses or cytotoxic reactions, particularly when they carry specific drugs such as small interfering RNA, necessitating stringent regulation to mitigate adverse reactions, especially in highly sensitive ear tissues [177, 178]. In the future, the damage to the integrity and biological activity of the carrier can be reduced by optimizing the process parameters, and the risk of cytotoxicity can be avoided through strict process standardization [178–180].

Beyond the challenges already discussed, current research is primarily reliant on animal models or in vitro experiments and therefore lacks robust evidence to support its translation to clinical applications. Furthermore, the precise molecular mechanisms governing exosomes-mediated regulation of ear diseases remain incompletely elucidated. While studies have indicated a potential role for exosomes in hearing damage repair, a systematic analysis of their interactions with inner ear cells and the specific key factors they deliver is currently lacking. This deficiency contributes to inconsistent interpretations of the underlying mechanisms. Future research should also focus on elucidating the precise protective or supportive mechanisms of exosomes derived from various sources, including plants, in the pathophysiology of ear disorders. Moreover, expanding exosomes research beyond murine models to include human in vitro samples is essential for a more comprehensive understanding of the role of exosomes in inner ear diseases and for facilitating the translation of research findings into clinical applications.

## Conclusion

Exosomes represent a promising avenue for both clinical diagnosis and therapy in otology. They carry specific proteins, microRNAs and lipids that can serve as valuable biomarkers, enabling early detection and continuous monitoring of disease progression. Beyond their diagnostic utility, exosomes also exhibit intrinsic therapeutic properties. Moreover, their ability to function as efficient and biocompatible delivery vehicles may significantly enhance treatment outcomes. Future research should focus on clarifying the mechanistic roles of exosomes in the pathogenesis of various ear diseases. In addition to the disorders discussed herein, exosomes-based strategies may also help address other challenging conditions, such as idiopathic sudden sensorineural hearing loss, genetic hearing impairment, and tinnitus. Further investigations are warranted to explore the application of exosomes as novel therapeutic modalities for ear diseases, particularly in the realms of targeted drug delivery and gene therapy. Concomitantly, the development of exosome-based diagnostic platforms represents a crucial and promising avenue for future research.

## Data Availability

The data and materials in this review are reasonably acquired from the corresponding author.

## Abbreviations

AAV: Adeno-associated virus
AB: β-amyloid
ABR: Auditory brainstem response
AD: Alzheimer
ADSCs: Adipose-derived stem cells
ARHL: Age-related hearing loss
BDNF: Brain-derived neurotrophic factor
BDNF-sEVs: BDNF-loaded sEVs
BLB: Blood-labyrinth barrier
CHP-EVs: Chondrocyte-homing peptide-modified extracellular vesicles
CSGPC-exos: Cochlear spiral ganglion progenitor cells
dUC: Ultracentrifugation
µDES: Droplet electroporation system
ESCRT: Endosomal sorting complex required for the transport
EVs: Extracellular vesicles
ERK: Extracellular Signal-Regulated Kinase
Exo-AAV: Exosome-associated AAV
GDN: Glia-derived nexin
hCPFs: human cholesteatoma perimatrix fibroblasts
HEI-OC1: House Ear Institute-Organ of Corti 1
HMEECs: Human middle ear epithelial cells
HMGB1: High-mobility group box 1
HSP70: Eat shock protein 70
I/R: Ischemia-Reperfusion
IL-1β: Interleukin-1 beta
IL-6: Interleukin-6
IL-10: Interleukin-10
IHCs: Inner hair cells
Ker-Exo: Keratinocyte-derived exosomes
MEE: Middle ear effusions
miR-21-Exo: miR-21-enriched exosomes derived from neural progenitor cells
MSC: Mesenchymal stem cells
MVBs: Multivesicular bodies
Myosin 7a: Myo7a
NIHL: Noise-induced hearing loss
NLRP3: NOD-like receptor family pyrin domain containing 3
OHCs: Outer hair cells
OIHL: Ototoxic drug-induced hearing loss
OM: Otitis media
PD: Parkinson
PDENs: Plant-derived exosome-like nanoparticles
PEG: Polyethylene glycol
RANKL: Receptor activator of nuclear factor-kB ligand
RNP: Ribonucleoprotein
SEC: Size-exclusion chromatography
sEVs: Small extracellular vesicles
SNHL: Sensorineural hearing loss
TB-EVs: Trophoblast-derived extracellular vesicles
TLR4: Toll-like receptor 4
UF: Ultrafiltration
